# Case report on pathogenetic link between gluten and IgA nephropathy

**DOI:** 10.1186/s12876-018-0792-0

**Published:** 2018-05-16

**Authors:** Stefano Costa, Giovanni Currò, Salvatore Pellegrino, Maria Cristina Lucanto, Giovanni Tuccari, Antonio Ieni, Giuseppina Visalli, Giuseppe Magazzù, Domenico Santoro

**Affiliations:** 1 Celiac Regional Centre, Pediatric Gastroenterology and Cystic Fibrosis Unit, University of Messina, Via Consolare Valeria 1, 98125 Messina, Italy; 2 Department of Human Pathology of Adult and Evolutive Age ‘Gaetano Barresi’, University of Messina, Messina, Italy; 3 Department of Biomedical and Dental Sciences and Morphofunctional Imaging, University of Messina, Messina, Italy; 4 Department of Clinical and Experimental Medicine, Unit of Nephrology and Dialysis, University of Messina, Messina, Italy

**Keywords:** IGA nephropathy, Celiac disease, Tissue transglutaminase, Immunofluorescence technique, Pathogenesis

## Abstract

**Background:**

A relationship between IgA nephropathy (IgAN) and celiac disease (CD) has been reported. We show the pathogenetic link for the first time.

**Case presentation:**

A 39-year-old man with cystic fibrosis (CF) and CF-related diabetes started to present gross hematuria, back pain and headache. At admission, laboratory analysis showed increase in serum creatinine of 1.5 mg/dl, together with hematuria and mild proteinuria (1 g/24 h). He underwent a renal biopsy to investigate the cause of hematuria and renal failure. Biopsy was consistent with IgAN. In view of patient reported dyspepsia, an upper gastrointestinal endoscopy with duodenal biopsies was undertaken and was normal. We looked for mucosal deposits of tTG-2 in the duodenum and the renal mesangium. tTG-2 deposits were found both in the duodenum and in renal biopsies, where they topographically replicated mesangial IgA deposits. After one year on a continued gluten containing diet, the patient developed a Marsh 2 type duodenal pathology.

**Conclusions:**

Our findings suggest a connection between CD and IgAN in terms of an immune-mediated gluten-induced pathogenesis even in the absence of villous atrophy and serum celiac autoantibodies.

**Electronic supplementary material:**

The online version of this article (10.1186/s12876-018-0792-0) contains supplementary material, which is available to authorized users.

## Background

A relationship between celiac disease and other autoimmune inflammatory disorders is known [1]. With regardto IgA nephropathy (IgAN), some studies report an increased incidence of celiac disease [2] and postulate a role of gluten in the pathogenesis of this disorder [3, 4]. A double immunofluorescence technique to detect intestinal deposits of anti-tissue transglutaminase IgA was successfully performed in patients with dermatitis herpetiformis (DH) [5] and gluten ataxia (GA) [6] showing a link between gluten and these diseases even in absence of duodenal atrophy or positive celiac serology.

We used this technique to detect anti-TG2 in the mesangium and duodenum of a patient with IgAN.

## Case presentation

**Fig. 1:**
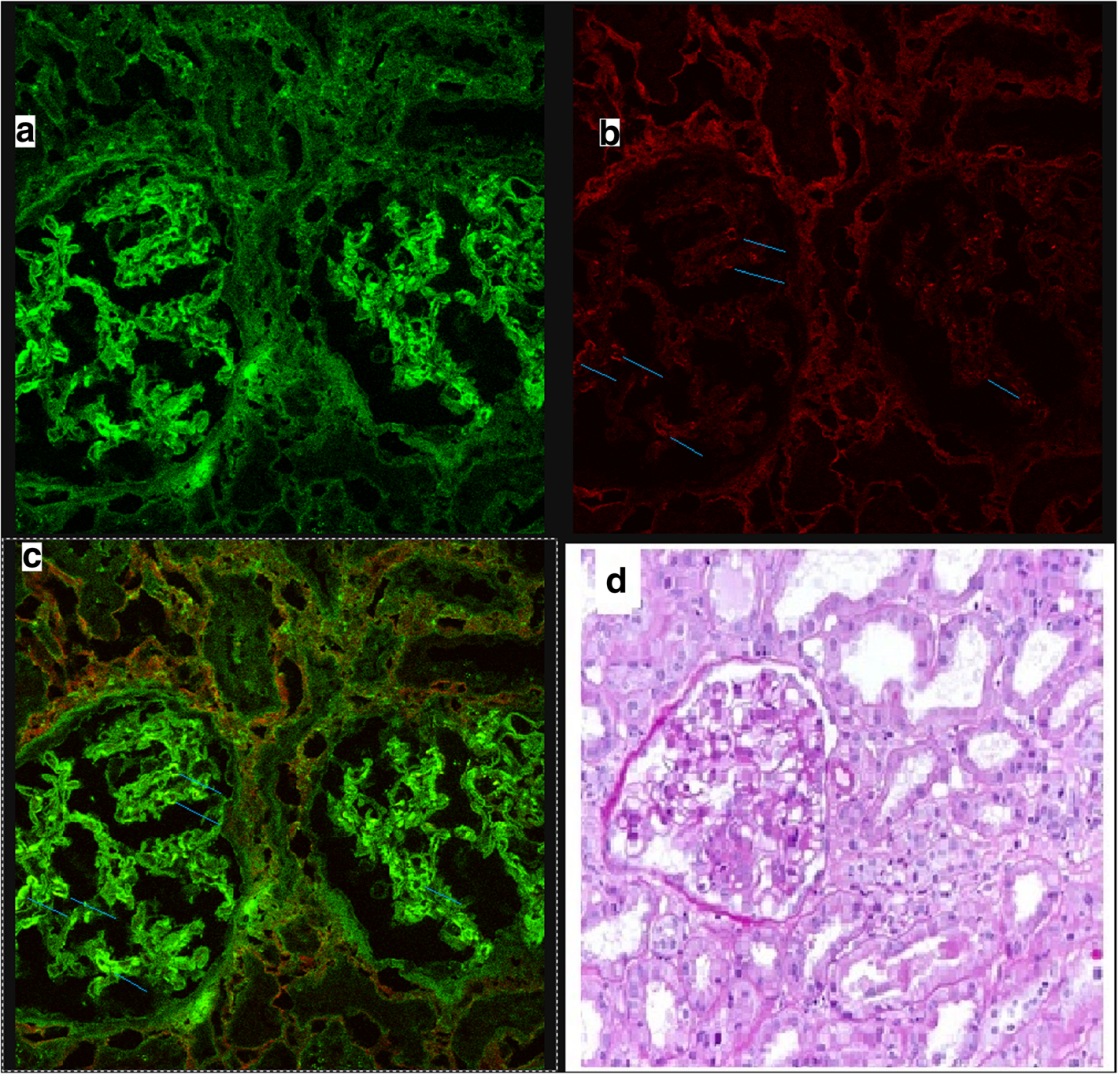
Renal biopsy. Immunofluorescence (**a**) showing mesangial deposition of IgA in green. In **b**, in red, deposition of TG2. In **c**, the overlap of red and green, in yellow, showing deposition of a-tTG2. The blue lines show the more intense deposition of IgA and TG2. In **d**, hematoxylin/eosin stain of renal biopsy

**Fig. 2:**
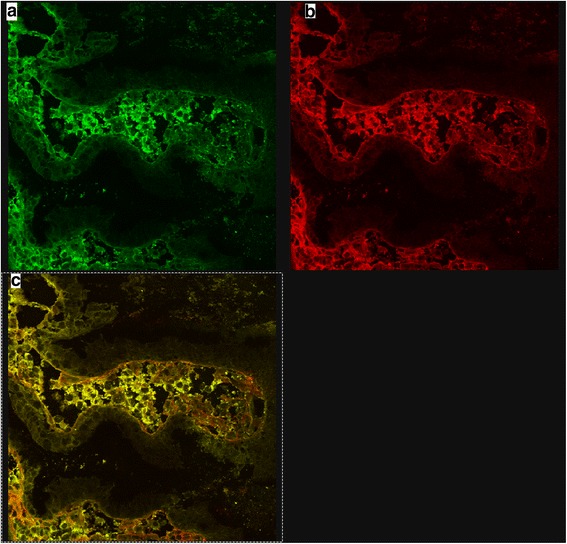
Duodenal biopsy. In **a**, deposition of IgA is showed in green. In **b**, deposition of TG2 is shown in red. In **c**, in yellow, the overlap of green and red demonstrating the presence of a-tTG2

A 39-year-old man with CF and CF-related diabetes came to our Unit due to symptoms of pulmonary exacerbation. At the same time, he started to present gross hematuria, back pain and headache. At admission, laboratory analysis showed increase in serum creatinine of 1.5 mg/dl together with hematuria and mild proteinuria (1 g/24 h). Intravenous antibiotics for pulmonary exacerbation (piperacillin/tazobacatam and colimycin) were started and a renal biopsy performed to investigate the cause of hematuria and renal failure. Biopsy was consistent with IgAN (see Fig. 1d), with morphological features of mesangial and endocapillary hypercellularity (Oxford classification M1, E1, S0, T0) [7] As the patient presented dyspepsia, he was offered upper gastrointestinal endoscopy with duodenal biopsies (one from duodenal bulb and three from descending duodenum). Duodenal mucosa was reported as normal on hematoxylin/eosin stain. While compatible HLA alleles for celiac disease (DQ2) were identified, both serum anti-endomysium (EMA) and anti-tissue transglutaminase antibodies (anti-tTG) were negative. It has been demonstrated that coeliac IgA targets intestinal TG2 early in disease development even when serum celiac autoantibodies are not present. Extraintestinal deposits of coeliac IgA further indicate that humoral immunity may have a pathogenetic role [8]. Therefore, double immunofluorescence on duodenal mucosa was performed to detect mucosal deposits of anti-tissue transglutaminase type 2 using the technique described by Karponay-Szabo et al., with some modification [8]. Briefly, 5 μm sections from the duodenal specimen included in optimal cutting temperature (OCT) compound were obtained and stored at − 80° in liquid nitrogen. Sections were fixed in acetone and incubated with Normal Rabbit Serum (Calbiochem Germany) for 20 min to block nonspecific sites. Sections were then incubated with anti-tissue transglutaminase type 2 (anti-tTG-2) from mouse (CUB7402 from Neomarker, Fremont CA) for one hour, and then with secondary antibodies conjugated with fluorochromes to detect total IgA (in green using Polyclonal Rabbit anti-Human IgA/FICT from Dako, Denmark) and anti-tTG-2 (in red using Polyconal Rabbit anti-Mouse RPE F (ab’) 2 from Dako, Denmark) for 30 min. The overlap of green and red (yellow) indicates the deposits of anti-tTG2. Analysis was performed on confocal microscopy. The same technique was performed on 5 μm renal sections. In both cases, an overlap of green and red in yellow was seen, thus demonstrating deposits of tTG-2 (Fig. 2a, b and c). In particular, in renal biopsies, deposits of tTG-2 that topographically replicated mesangial deposits of IgA (Fig. 1) were detected.

Given the morphological features of IgAN, and the presence of proteinuria, steroid therapy was started according to a standardized six month’s protocol [9]. After one month, proteinuria decreased to 100 mg/24 h and hematuria resolved. Steroids were stopped after six months of treatment. The patient continued on a gluten containing diet.

**Fig. 3:**
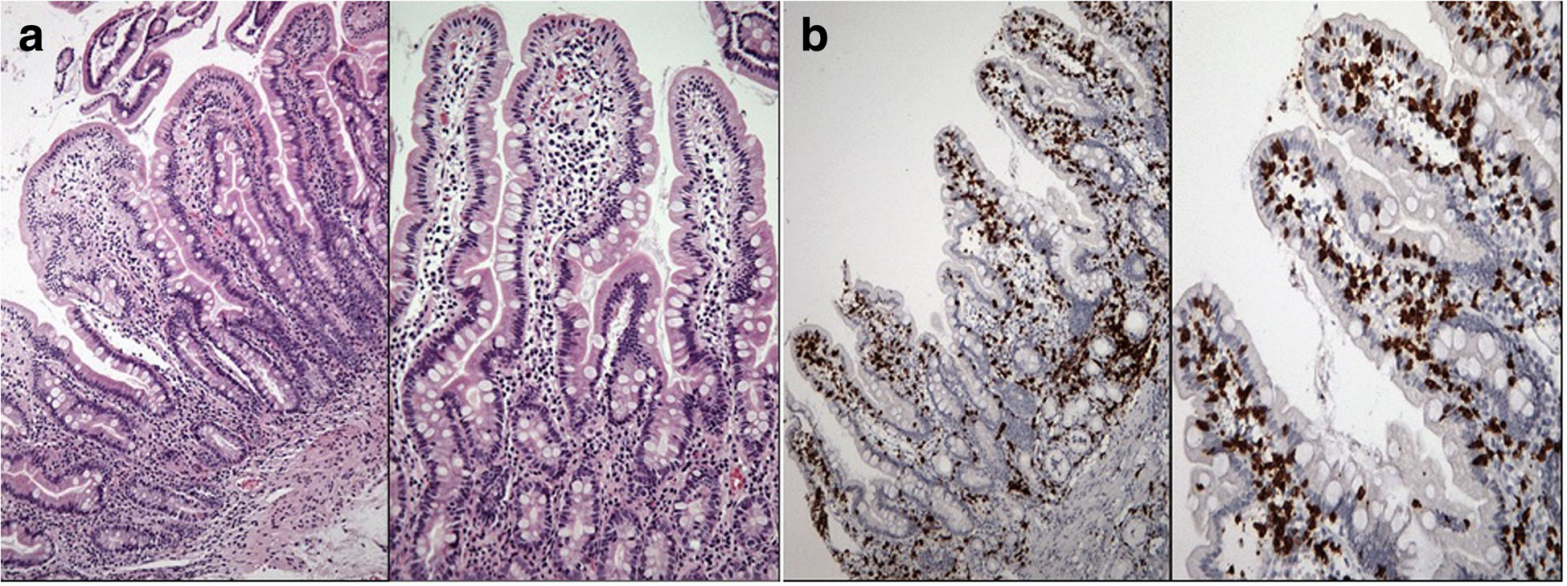
Duodenal biopsy after one year. In **a**, hematoxylin/eosin stain showing cryptic hyperplasia. In **b**, the CD3 immunostained section for the count of intraepithelial lymphocytes

Considering that intestinal deposits of anti-tissue transglutaminase IgA are predictive of forthcoming enteropathy [10] after one year arepeat upper endoscopy was performed even though anti-tTG were still negative. At this time, a Marsh 2 type duodenal pathology was detected (Fig. 3a, b). A gluten free diet was started (for a complete timeline history see Additional file 1: Table S1).

## Discussion and conclusions

To our knowledge, this is the first demonstration of the pathogenetic role of gluten in IgAN. IgAN is a rare complication of CF [11]. In these cases, it is supposed that aninfectious stimulus acts as a trigger. On the other hand, a relationship between CD and IgAN has previously been suggested. In 2002, Collin et al. demonstrated an incidence of 3.6% of biopsy proven CD in IgAN [2]. On the other hand, a recent study demonstrated an increased risk of biopsy-verified IgAN among individuals with CD [12]. Another link between these two diseases is represented by the ability of gliadin to act as a lectin and to form immunocomplexes with IgA1N (oligosaccharide-containing IgA). Coppo et al. were able to induce experimental IgAN in mice immunized with gliadin [3]. The same authors showed a positive effect of a gluten free diet on proteinuria and on progression of renal failure in patients with IgAN. Berthelot et al. showed how activation of transglutaminase 2 is crucial for the deposition of immune-complexes in the mesangium of patients with IgAN [13]. Lechner et al. showed that the removal of IgA deposits by a proteases led to a decrease of TG2 staining thus confirming the presence of the enzyme in mesangial immunocomplexes [14].

In several case reports, an improvement of IgAN is described following a gluten free diet [15–17]. All these cases are characterized by the presence of a biopsy proven CD and in no caseis a contemporary immune activation in intestine and kidney reported before the development of duodenal atrophy.

Our finding can explain how CD and IgAN are related. As we found deposits of IgA anti-tissue transglutaminase both in the duodenum and glomerular mesangium we can argue that an autoimmune activation, triggered by gluten in intestinal mucosa and involving tissue transglutaminase, mayoccur in extraintestinal target sites. This kind of association has been demonstrated in other diseases such as GA and in DH, where the role of gluten is well recognized, and a gluten free diet is beneficial [5, 6]. Duodenal atrophy can be absentalso in these diseases, as auto-antibodies production is directed against specific transglutaminase isotypes represented, in dermis, by isotype 3, and in myelin by isotype 6, in DH and GA, respectively, In particular, in GA a contemporary presence of anti-tTG2 deposits was found in jejunal mucosa and within the muscular layer of brain vessels and brain parenchyma particularly of the cerebellum, regardless of the presence of duodenal atrophy or positive celiac serology. In our case the strength of pathogeneticassociation between CD and IgAN depends on the diagnostic power of the technique we adopted to detect a gluten dependent immune activation. Duodenal deposits oftTG2 are very sensitive and specific for diagnosis of celiac disease, even in the absence of duodenal atrophy and positive celiac serology [18]. Moreover, Salmi et al. [10] showed how mucosal anti-tTG2 deposits strongly predict forthcoming duodenal atrophy.

Actually, after one year, the patient developed a duodenal pathology, thus confirming that intestinal deposits of anti-tTG2 were the initial manifestation of a gluten driven inflammation. As transglutaminase 2 is the most represented isoform in the mesangium, we suggest that mesangial deposition of anti-tTG-2 may play a determinant role in pathogenesis of the disease even in the absence of other signs suggesting celiac disease.

Our findings suggest an immune-mediated gluten-induced pathogenetic link between CD and IgAN even in the absence of villous atrophy and serum celiac autoantibodies. Searching for mesangial deposits of anti-tTG2 in a large series of new cases of IgAN will allow to define how many cases are caused by this autoimmune mechanism and if a gluten free diet can really improve renal outcome of these patients.

## Additional file

Additional file 1: **Table S1.** Medical history timeline. (PDF 3430 kb)

## Abbreviations

CD: Celiac disease
CF: Cystic fibrosis
DH: Dermatitis herpetiformis
EMA: Anti-endomysium
GA: Gluten ataxia
IgAN: IgA nephropathy
IgA-tTG: IgA-tissue antitransglutaminase antibodies
