# Supplementary material for: Case report on pathogenetic link between gluten and IgA nephropathy
Source: BMC Gastroenterol. 2018 May 16;18:64. doi: 10.1186/s12876-018-0792-0 (PMC5956757; doi:10.1186/s12876-018-0792-0)
Supplement: Supplementary file 1 — Table S1. Medical history timeline. (PDF 3430 kb) [file 12876_2018_792_MOESM1_ESM.pdf]

Table S1. Medical history timeline

Date	Medical history	Diagnostic procedures	Therapeutic interventions
**1/2014**	gross hematuria, back pain and headache during pulmonary exacerbation of cystic fibrosis	Renal biopsy , IgAN diagnosed (mesangial deposits of anti-tTG2 detected)	Intravenous antibiotics for pulmonary exacerbation (piperacillin/tazobacatam and colimycin)
**1/2014**	dyspepsia	upper gastrointestinal endoscopy with duodenal biopsies (intestinal deposits of anti-tTG2 detected)	Gluten containing diet
**1/2014**			Six months steroid therapy protocol for IgAN
**7/2014**	Clinical remission of IgAN		Steroid withdrawal
**1/2015**		upper gastrointestinal endoscopy with duodenal biopsies (Marsh 2 type duodenal damage was detected)	Gluten free diet started
